# Effect of dexmedetomidine on inflammation in patients with sepsis requiring mechanical ventilation: a sub-analysis of a multicenter randomized clinical trial

**DOI:** 10.1186/s13054-020-03207-8

**Published:** 2020-08-10

**Authors:** Yoshinori Ohta, Kyohei Miyamoto, Yu Kawazoe, Hitoshi Yamamura, Takeshi Morimoto

**Affiliations:** 1 Education and Training Center for Students and Professionals in Healthcare, Hyogo College of Medicine, Nishinomiya, Japan; 2 Department of Emergency and Critical Care Medicine, Wakayama Medical University, Wakayama, Japan; 3 Division of Emergency and Critical Care Medicine, Tohoku University Graduate School of Medicine, Sendai, Japan; 4 Osaka Prefectural Nakakawachi Emergency and Critical Care Center, Higashiosaka, Japan; 5 Department of Clinical Epidemiology, Hyogo College of Medicine, Nishinomiya, Japan

**Keywords:** Dexmedetomidine, Inflammation, Sepsis, C-reactive protein, Procalcitonin

## Abstract

**Background:**

Administration of dexmedetomidine has been reported to improve inflammatory response in animals. We explored the effects of administering dexmedetomidine on the levels of C-reactive protein (CRP) and procalcitonin, and thus on inflammation, in patients with sepsis enrolled in a randomized clinical trial.

**Methods:**

The DESIRE trial was a multicenter randomized clinical trial in which adult patients with sepsis were sedated with (DEX group) or without (non-DEX group) dexmedetomidine while on mechanical ventilators. As a prespecified sub-analysis, we compared CRP and procalcitonin levels during the first 14 days of treatment between the two groups. The 14-day mortality rate, albumin level, and the number of patients with disseminated intravascular coagulation (DIC) were also assessed. We used generalized linear models to estimate the differences in these outcomes between groups. We also used the Kaplan-Meier method to estimate the 14-day mortality rate and the log-rank test to assess between-group differences.

**Results:**

Our study comprised 201 patients: 100 in the DEX group and 101 in the non-DEX group. CRP and procalcitonin levels were lower in the DEX vs. non-DEX group during the 14-day treatment period [CRP—range, 5.6–20.3 vs. 8.3–21.1 mg/dL (*P* = 0.03); procalcitonin—range, 1.2–37.4 vs. 1.7–52.9 ng/mL (*P* = 0.04)]. Albumin levels were higher in the DEX group (range, 2.3–2.6 g/dL) than in the non-DEX group (range, 2.1–2.7 g/dL; *P* = 0.01). The percentage of patients with DIC did not significantly differ between the groups (range, 21–59% and 17–56% for the DEX and non-DEX groups, respectively; *P* = 0.49). The 14-day mortality rates in the DEX and non-DEX groups were 13 and 21%, respectively (*P* = 0.16).

**Conclusion:**

Sedation using dexmedetomidine reduced inflammation in patients with sepsis requiring mechanical ventilation.

**Trial registration:**

ClinicalTrials.gov, NCT01760967. Registered on 4 January 2013.

## Background

The inflammatory response has catastrophic consequences in patients with sepsis, including multiple organ failure and death [1]. Leukocytes secrete inflammatory mediators such as interleukin (IL)-6 and tumor necrosis factor (TNF)-α during critical illness. These mediators can cause endothelial dysfunction, which affects vascular permeability, as well as thrombin activation, leading to hypotension, metabolic acidosis, tissue damage, and eventual organ failure [1, 2]. IL-6 and TNF-α concurrently stimulate the production of C-reactive protein (CRP) and procalcitonin (PCT), which are the most widely used inflammatory biomarkers in patients with sepsis in clinical practice [3, 4]. Although CRP is a marker of the acute inflammatory response, rather than of the infection that caused the sepsis, it is used to assess the severity and progression of sepsis-induced inflammation [3]. PCT levels rise in response to a variety of pro-inflammatory stimuli, especially those of bacterial origin. They closely correlate with the severity of systemic inflammation and can be used to monitor the course of sepsis [4, 5].

Dexmedetomidine is a highly selective α_2_-adrenergic agonist widely used for sedation of patients during mechanical ventilation [6]. Several studies have shown that it also suppresses the inflammatory response [7–9]. Administration of dexmedetomidine decreases IL-6 and TNF-α levels in animals with severe inflammation [7, 8] and IL-6, TNF-α, and CRP levels in humans undergoing surgery [9]. Whether it also reduces inflammation in patients with sepsis is not known.

To investigate the effect of administering dexmedetomidine on sepsis-induced inflammation, we compared the outcomes of patients with sepsis who were treated with or without dexmedetomidine for 14 days as part of the DESIRE clinical trial [10].

## Methods

### Study design

This analysis was prespecified in the study protocol for the DESIRE trial before patient enrollment began. The DESIRE trial was a multicenter, open-label, randomized clinical trial that compared the sedation strategies of administering dexmedetomidine (DEX group) versus not administering dexmedetomidine (non-DEX group) in terms of mortality and ventilator-free days during a 28-day period [10]. The trial was registered at ClinicalTrials.gov (identifier: NCT 01760967). A total of 201 patients were enrolled from eight intensive care units (ICUs) in Japan between February 2013 and January 2016, all of whom had sepsis requiring mechanical ventilation for more than 24 h [10]. The flow of participants through the trial is shown in the Supplemental Figure (see Additional File 1). In the trial, sepsis was defined as systemic inflammatory response syndrome (SIRS) criteria due to infection [11]. The enrolled patients satisfied the sepsis-III criteria due to having received mechanical ventilation and having a Sequential Organ Failure Assessment (SOFA) score of 2 or more [12].

Patients in the DEX and non-DEX groups received other sedatives as needed to achieve the sedation target and analgesics if necessary. In both groups, the targets of sedation depth were a Richmond Agitation-Sedation Scale score of 0 (calm) during the day and − 2 (lightly sedated) during the night [13]. The treatment protocol for sepsis was based on the guidelines developed by the Japanese Society of Intensive Care Medicine for sepsis management [14]. The present study was approved by the institutional review boards at each participating center. Additional study protocols, inclusion and exclusion criteria, and informed consent are described in the Supplemental Methods (see Additional File 1). To evaluate the acute phase of inflammation, this sub-analysis compared the inflammatory status of the DEX and non-DEX groups during the first 14 days after the randomization.

### Outcome measurements

The primary outcome in this analysis was inflammation as indicated by CRP and PCT levels. The secondary outcomes were 14-day mortality, albumin (ALB) level, and sepsis-associated coagulopathy. The ALB level served as a marker of vascular permeability [15]. The disseminated intravascular coagulation (DIC) score, as defined by the Japanese Association for Acute Medicine, was used to assess sepsis-associated coagulopathy [16]. The DIC score comprises platelet (PLT) counts, the patient/normal prothrombin time (PT) ratio, the levels of fibrin/fibrinogen degradation products (FDPs), and the number of SIRS characteristics. The scoring system for DIC is summarized in the supplemental table 1 (see Additional file 1). CRP, PCT, ALB, and FDP levels, PLT counts, and PT ratios were measured throughout the 14-day observation period. We also determined the number of patients with three or more SIRS characteristics and/or DIC (as based on the DIC score) on days 1, 2, 4, 6, 8, 10, 12, and 14.

### Statistical analysis

Primary and secondary outcomes were analyzed according to the intention-to-treat principle. Continuous variables were presented as means with standard deviations (SDs) or medians with interquartile ranges, and categorical variables were presented as numbers and percentages. To compare patient characteristics between the groups, we used *t* tests or Wilcoxon rank sum tests for continuous variables and chi-square tests or Fisher’s exact tests for categorical variables.

We used a generalized linear model (the GENMOD procedure) to examine the effect of administering dexmedetomidine on CRP, PCT, ALB, and FDP levels, PLT counts, and PT ratios and to account for repeated measurements in the same patient. We used a generalized linear model (the GENMOD procedure with logit) to determine the effect of administering dexmedetomidine on the number of patients with three or more SIRS characteristics and/or DIC. The variables describing patient status were the dependent variables, and treatment allocation was the independent variable with a repeated variable of patient. The cumulative incidence of mortality over the 14-day treatment period was estimated via the Kaplan-Meier method, and differences between the groups were assessed using the log-rank test.

All statistical analyses were performed using JMP version 11.2.0 (SAS Institute Inc.), and SAS version 9.4 (SAS Institute Inc.) software. A two-sided *P* value < 0.05 was considered statistically significant.

## Results

Among the 201 patients in our study, 127 (63%) were men, and the mean age was 69 years (SD, 14 years) (Table 1). The median Acute Physiology and Chronic Health Evaluation II score was 23, and the median SOFA score was 9. There were 100 patients in the DEX group and 101 in the non-DEX group; the characteristics of the groups were well balanced. On the first day after randomization, the median and highest CRP levels were 13.8 and 48.3 mg/dL in the DEX group and 16.8 and 44.0 mg/dL in the non-DEX group, respectively. The number of patients with a PCT level of more than 0.5 ng/mL was 88 (89%) in the DEX group and 88 (90%) in the non-DEX group. The mean ALB level was 2.6 g/dL in the DEX group and 2.7 g/dL in the non-DEX group; the number of patients with hypoalbuminemia (ALB of 3.5 mg/dL or less) was 90 (90%) in the DEX group and 89 (88%) in the non-DEX group. Forty (40%) patients in the DEX group and 42 (42%) in the non-DEX group had DIC on day 1.

**Table 1:** Patient characteristics

Characteristic	DEX group (*n* = 100)	Non-DEX group (*n* = 101)	*P* value
Age (years), mean (SD)	68 (14.9)	69 (13.6)	0.75
Men, n (%)	63 (63)	64 (63)	0.96
Body weight (kg), mean (SD)	55 (12.5)	58 (15.3)	0.09
COPD, *n* (%)	8 (8)	9 (9)	0.82
Soft tissue infection, *n* (%)	8 (8)	10 (10)	0.64
Emergency surgery, *n* (%)	37 (37)	36 (36)	0.84
Site of infection, *n* (%)			
Abdomen	39 (39)	35 (35)	0.52
Thorax	39 (39)	33 (33)	0.35
Urinary tract	6 (6)	10 (10)	0.31
Pancreatitis	3 (3)	9 (9)	0.13
Skin and soft tissue	6 (6)	7 (7)	0.79
Central nervous system	1 (1)	1 (1)	1.00
Others	6 (6)	6 (6)	0.99
APACHE II score, median [IQR]	23 [18, 29]	22 [16, 29.5]	0.51
SOFA score, median [IQR]	8 [6, 11]	9 [5, 11]	0.67
SOFA respiratory score, median [IQR]	2 [1, 3]	2 [1, 3]	0.86
SOFA circulatory score, median [IQR]	3 [2, 4]	3 [1.5, 4]	0.44
SOFA renal score, median [IQR]	1 [0, 2]	1 [0, 3]	0.28
SOFA hepatic score, median [IQR]	0 [0, 1]	0 [0, 1]	0.42
SOFA neurological score, median [IQR]	1 [0, 2]	0 [0, 3]	0.87
SOFA coagulation score, median [IQR]	0 [0, 2]	1 [0, 2]	0.26
CRP (mg/dL), median [IQR]	13.8 [6.0, 25.7]	16.8 [6.4, 25.8]	0.64
PCT (ng/mL), median [IQR]	15.2 [2.8, 44.9]	14.6 [1.5, 81.5]	0.70
ALB (g/dL), mean (SD)	2.6 (0.7)	2.7 (0.7)	0.64
PLT (10^9^/L), median [IQR]	170 [98, 227]	141 [95, 224]	0.34
PT (INR), median [IQR]	1.21 [1.06, 1.37]	1.24 [1.08, 1.45]	0.34
FDPs (mcg/mL), median [IQR]	16.0 [7.8, 29.3]	17.4 [8.8, 33.5]	0.80
SIRS criteria ≥ 3, *n* (%)	71 (71)	75 (74)	0.60
DIC, *n* (%)	40 (40)	42 (42)	0.87

Abbreviations: *DEX* dexmedetomidine, *COPD* chronic obstructive pulmonary disease, *APACHE II* Acute Physiology and Chronic Health Evaluation II, *IQR* interquartile range, *SOFA* Sequential Organ Failure Assessment, *SD* standard deviation, *CRP* C-reactive protein, *PCT* procalcitonin, *ALB* albumin, *PLT* platelet, *PT-INR* prothrombin time-international ratio, *FDPs* fibrin/fibrinogen degradation products, *SIRS* systemic inflammatory response syndrome, *DIC* disseminated intravascular coagulation

**Fig. 1:**
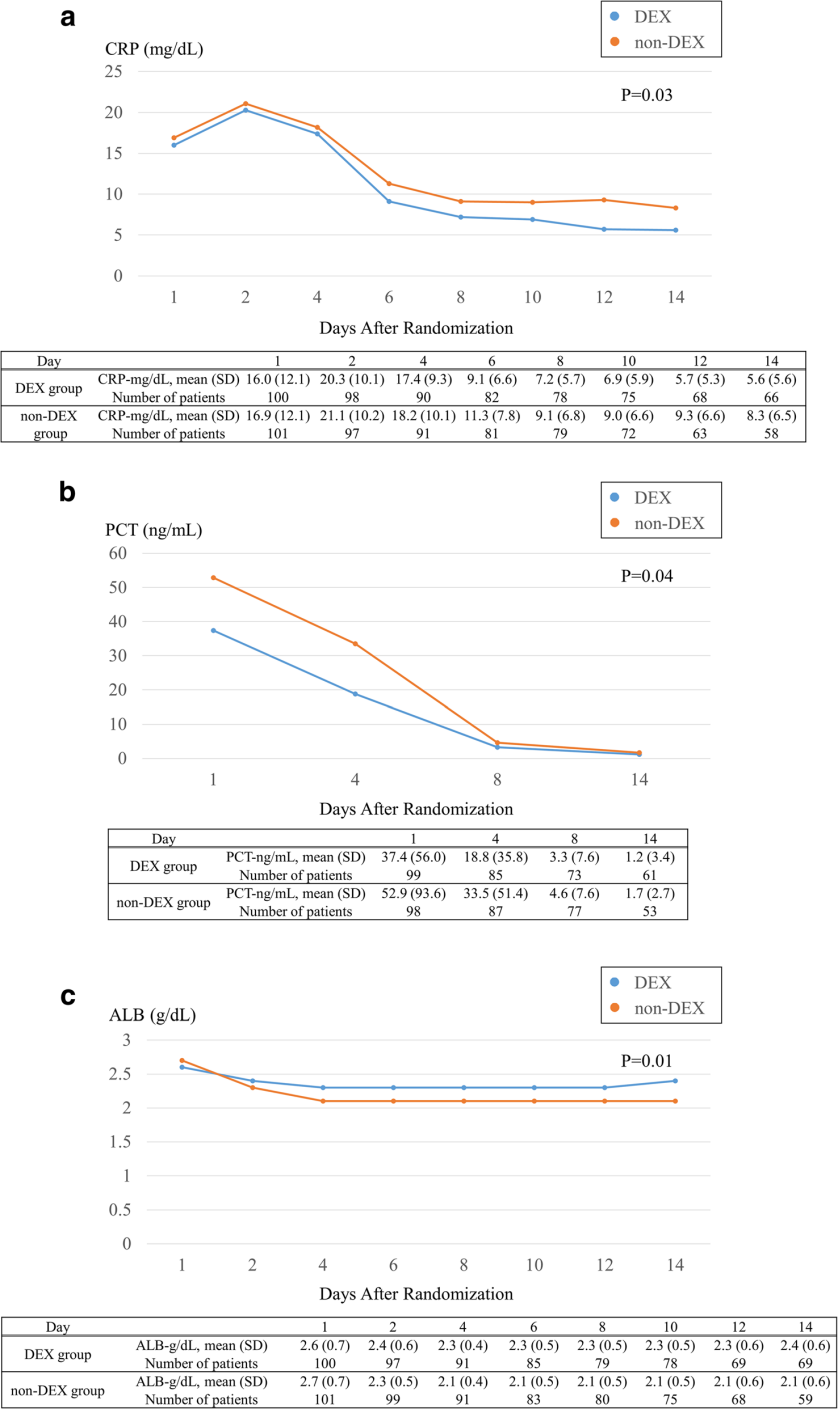
Changes in outcome measurements in patients with sepsis treated with or without dexmedetomidine (DEX) for 14 days. **a** C-reactive protein (CRP). **b** Procalcitonin (PCT). **c** Albumin (ALB). SD, standard deviation

CRP and PCT levels in the DEX and non-DEX groups during the 14-day observation period are shown in Fig. 1a and b. The CRP level was highest on day 2 in both the DEX (20.3 mg/dL) and the non-DEX (21.1 mg/dL) groups (Fig. 1a). It was significantly lower in the DEX group (range, 5.6–20.3 mg/dL) than in the non-DEX group (range, 8.3–21.1 mg/dL) over the 14-day observation period (*P* = 0.03). PCT levels were measured on days 1, 4, 8, and 14 and were highest on day 1 in both the DEX (37.4 ng/mL) and non-DEX (52.9 ng/mL) groups (Fig. 1b). The PCT level was significantly lower in the DEX group (range, 1.2–37.4 ng/mL) than in the non-DEX group (range, 1.7–52.9 ng/mL) over the 14-day observation period (*P* = 0.04).

The ALB levels in the DEX and non-DEX groups during the 14 days after randomization are shown in Fig. 1c. The ALB level was lowest on days 4–12 in the DEX group and on days 4–14 in the non-DEX group. ALB levels were reduced to a lesser extent in the DEX group (difference, − 0.3 g/dL) than in the non-DEX group (difference, − 0.6 g/dL). The ALB level was significantly higher in the DEX group (range, 2.3–2.6 g/dL) than in the non-DEX group (2.1–2.7 g/dL) over the 14-day treatment period (*P* = 0.01).

The levels of the coagulation biomarkers and the number of patients with three or more SIRS characteristics during the 14-day observation period are shown in the Supplement Table 2 (see Additional File 1). PLT counts were lowest on day 4 in both groups and overall did not differ significantly between groups (range, 116–317 × 10^9^/L in the DEX group and 104–299 × 10^9^/L in the non-DEX group; *P* = 0.72). FDP levels were also similar in both groups (range, 22.9–34.3 mcg/mL in the DEX group and 19.1–51.7 mcg/mL in the non-DEX group; *P* = 0.40). The PT ratio was highest in both groups on day 2 but overall was lower in the DEX group (range, 1.13–1.38) than in the non-DEX group (1.21–1.48; *P* = 0.03). The percentage of patients with three or more SIRS characteristics did not differ significantly between the DEX vs. non-DEX group (range, 12–71 vs. 19–74%; *P* = 0.15). The levels of the DIC-associated variables were similar in both groups with the exception of the PT ratio (Supplement Table 2). The percentage of patients with DIC was also similar in both groups (range, 21–59% in the DEX group and 17–59% in the non-DEX group; *P* = 0.49).

**Fig. 2:**
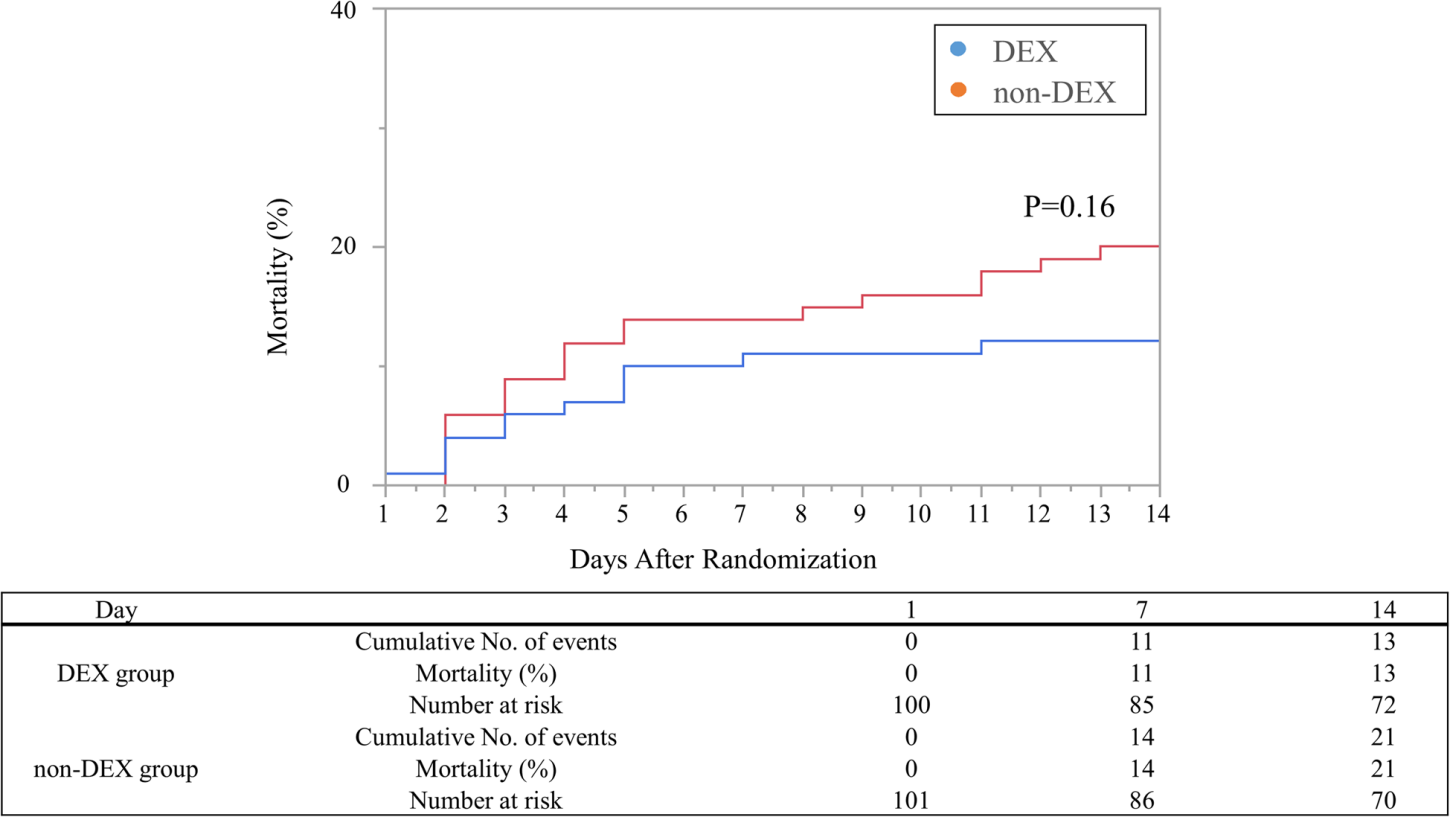
The 14-day mortality rates for the DEX and non-DEX groups

Among the 201 patients in our study, 34 (17%) died during the 14 days after randomization. The mortality rates in the DEX and non-DEX groups were 13% (13 patients) and 21% (21 patients), respectively (*P* = 0.16) (Fig. 2).

## Discussion

We analyzed data derived from a randomized clinical trial and found that the administration of dexmedetomidine to patients with sepsis on ventilators improved CRP and PCT levels during the first 14 days in the ICU. It also reduced the incidence of hypoalbuminemia, but not of DIC. The 14-day mortality rate was also 8% lower in the DEX group than in the non-DEX group, but this reduction was not significant. The original DESIRE trial did not find statistically significant superiority of administering dexmedetomidine in terms of mortality, but the findings implied an 8% reduction in the 28-day mortality rate in patients with sepsis [10]. Because the different use of sedatives did not account for the reduction of mortality, the mechanism of the effect of administering dexmedetomidine on mortality should be explored.

Dexmedetomidine is a unique sedative: unlike aminobutyric acid receptor agonists, it has analgesic [6] and anti-inflammatory [7–9] effects. The latter may reflect its ability to inhibit the expression of inflammatory molecules when bound to α_2_-adrenergic receptors on macrophages [17] and/or to increase the concentration of norepihephrine (which suppresses the immune response) when bound to synaptic α_2_-adrenergic receptors in the central nervous system [18–20]. Other possible mechanisms include the modulation of cytokine production by macrophages and monocytes; the inhibition of apoptosis and the Toll-like receptor 4 and myeloid differentiation factor 88/mitogen-activated protein kinase/nuclear factor-κB signaling pathways; and the stimulation of the cholinergic anti-inflammatory pathway [19–24].

Although the anti-inflammatory mechanisms through which the administration of dexmedetomidine suppresses inflammation remain to be fully elucidated, previous studies suggest that administration of dexmedetomidine decreases the levels of pro-inflammatory cytokines such as IL-6 and TNF-α in patients with sepsis [25], as well as the levels of pro-inflammatory cytokines and CRP in patients undergoing surgery while under anesthesia [9]. In the present study, the use of dexmedetomidine for sedation reduced both CRP and PCT levels in patients with sepsis. This result indicates that the administration of dexmedetomidine alleviates infection-induced inflammation.

We also aimed to determine whether administration of dexmedetomidine improved hypoalbuminemia and sepsis-associated coagulopathy, both of which are associated with severe inflammation. Systemic inflammation increases vascular permeability, which is a major cause of hypoalbuminemia in patients with sepsis [2]. Most of the patients in our study had low albumin levels on the first day after randomization. Thereafter, however, albumin levels were higher in patients treated with versus without dexmedetomidine. This finding presumably reflects reduced inflammation rather than improved nutrition: during the acute phase of inflammation, levels of albumin, which has a half-life of 21 days, are more influenced by vascular permeabilization caused by inflammation-induced injuries to the endothelium than by nutrition [26, 27]. Sepsis-associated coagulopathy, which can lead to DIC, is thought to result from crosstalk between the inflammation and coagulation systems; inflammation triggers coagulation, which in turn promotes inflammation [28, 29]. In this study, the administration of dexmedetomidine did not affect the incidence of coagulation or DIC. Suppression of inflammation by administering dexmedetomidine might be insufficient for preventing coagulopathy.

The administration of dexmedetomidine has been shown to reduce not only plasma inflammatory cytokine concentrations but also mortality in rats with endotoxin-induced shock, and these effects were reported to be dose-dependent [30]. A recent database study reported that mortality differed according to the time after diagnosis of sepsis; phases were categorized in that study as phase 1 (days 1 to 5), phase 2 (days 6 to 15), and phase 3 (days 16 to 150) [31]. Deaths during phase 1 and phase 2 accounted for 67.5% of mortality in patients with sepsis or septic shock [31], and one cause of the early peak in mortality was severe inflammation [1]. Therefore, we focused on both inflammation status and mortality in a 14-day period. CRP levels correlate positively with the risk of organ failure and mortality; hence, monitoring these levels may aid in assessing the response to therapy in patients with sepsis [32, 33]. PCT has greater diagnostic accuracy than does CRP because it differentiates infectious versus non-infectious causes of inflammation [34]. CRP is a risk factor for mortality in patients with sepsis when levels are elevated and the clearance rate is low [35]. Hypoalbuminemia has been shown to predict 30-day mortality in critically ill patients [36].

The doses of dexmedetomidine ranged from 0.1 to 0.7 mcg/kg/h in this study; these are the standard approved doses in Japan. Although these doses are lower than those typically administered in Western countries, the administration of dexmedetomidine at these lower doses successfully decreased CRP and PCT levels and increased albumin levels when administered to patients with sepsis for 14 days. These changes were considered to reflect the improvement of inflammation in the first 14 days after sepsis onset; hence, it might be associated with decreased mortality. As noted above, a point estimate of 8% reduction in mortality at 14 day was observed in our study, although a level of statistical significance was not reached. One possible reason for this non-significant reduction in mortality could be due to the relatively small sample size or the lower doses of dexmedetomidine. As the anti-inflammatory effects of administering dexmedetomidine are reportedly dose-dependent [30], a greater reduction in mortality might be observed if higher doses are used. Further research should clarify the effect of administering dexmedetomidine with higher doses on patient mortality and potential adverse effects in patients with sepsis.

There are two limitations in this study. First, it was an open-label study, and the endpoints were assessed by a physician at discharge. Awareness of the treatment assignment (dexmedetomidine or no dexmedetomidine) may have influenced some of the management protocols. However, CRP, PCT, and ALB levels, 14-day mortality rates, and sepsis-associated coagulopathy would not be affected by the judgments of physicians. Although preparations containing ALB can increase albumin levels, the physicians in charge followed the guidelines regarding such preparations when treating patients with sepsis-associated hypoalbuminemia [14]. Second, because our study was a subcomponent of the DESIRE trial, sample sizes with sufficient power to detect clinically meaningful differences were not calculated and thus may have been inadequate in some cases, especially in those involving sepsis-associated coagulopathy. Moreover, multiple endpoints in sub-analyses should be treated with caution owing to inflated alpha errors. However, the consistency of our findings (lower CRP and PCT levels and higher ALB level in the DEX group) attest to their validity.

## Conclusions

The administration of dexmedetomidine significantly improved CRP, PCT, and ALB levels in patients with sepsis requiring mechanical ventilation. Because the anti-inflammatory effects of administering dexmedetomidine were not associated with the reduction of mortality in patients with sepsis at 14 days, further studies with larger sample sizes or administering higher doses of dexmedetomidine are warranted.

## Supplementary information

**Additional file 1: Supplemental Methods. Table S1.** Scoring system for DIC. **Table S2.** Changes in DIC and DIC-associated variables. **Figure S1.** Flow of Participants in the DESIRE trial.

## Data Availability

The data that support the findings of this study are available from the corresponding author upon reasonable request.

## Abbreviations

IL: Interleukin
TNF: Tumor necrosis factor
CRP: C-reactive protein
PCT: Procalcitonin
ICU: Intensive care units
SIRS: Systemic inflammatory response syndrome
SOFA: Sequential Organ Failure Assessment
ALB: Albumin
DIC: Disseminated intravascular coagulation
PLT: Platelet
PT: The patient/normal prothrombin time
FDPs: Fibrin/fibrinogen degradation products
SD: Standard deviation

## References

[1] Delano MJ, Ward PA (2006). The immune system’s role in sepsis progression, resolution, and long-term outcome. Immunol Rev.

[2] Wheeler AP, Bernard GR (1999). Treating patients with severe sepsis. N Engl J Med.

[3] Povoa P (2002). C-reactive protein: a valuable marker of sepsis. Intensive Care Med.

[4] Nijsten MW, Olinga P, de Vries EG, Koops HS, Groothuis GM, Limburg PC, ten Duis HJ, Moshage H, Hoekstra HJ, Bijzet J, Zwaveling JH, The TH (2000). Procalcitonin behaves as a fast responding acute phase protein in vivo and in vitro. Crit Care Med.

[5] Wacker C, Prkno A, Brunkhorst FM, Schlattmann P (2013). Procalcitonin as a diagnostic marker for sepsis: a systematic review and meta-analysis. Lancet Infect Dis.

[6] Venn RM, Bradshaw CJ, Spencer R, Brealey D, Caudwell E, Naughton C, Vedio A, Singer M, Feneck R, Treacher D, Willatts SM, Grounds RM (1999). Preliminary UK experience of dexmedetomidine, a novel agent for postoperative sedation in the intensive care unit. Anaesthesia.

[7] Xu L, Bao H, Si Y, Wang X (2013). Effects of dexmedetomidine on early and late cytokines during polymicrobial sepsis in mice. Inflamm Res.

[8] Taniguchi T, Kidani Y, Kanakura H, Takemoto Y, Yamamoto K (2004). Effects of dexmedetomidine on mortality rate and inflammatory responses to endotoxin-induced shock in rats. Crit Care Med.

[9] Li Y, He R, Chen S, Qu Y (2015). Effect of dexmedetomidine on early postoperative cognitive dysfunction and peri-operative inflammation in elderly patients undergoing laparoscopic cholecystectomy. Exp Ther Med.

[10] Kawazoe Y, Miyamoto K, Morimoto T, Yamamoto T, Fuke A, Hashimoto A, Koami H, Beppu S, Katayama Y, Itoh M, Ohta Y, Yamamura H (2017). Effect of dexmedetomidine on mortality and ventilator-free days in patients requiring mechanical ventilation with sepsis: a randomized clinical trial. JAMA.

[11] Bone RC, Balk RA, Cerra FB, Dellinger RP, Fein AM, Knaus WA, Schein RM, Sibbald WJ (1992). Definitions for sepsis and organ failure and guidelines for the use of innovative therapies in sepsis. The ACCP/SCCM Consensus Conference Committee. American College of Chest Physicians/Society of Critical Care Medicine. Chest.

[12] Singer M, Deutschman CS, Seymour CW, Shankar-Hari M, Annane D, Bauer M, Bellomo R, Bernard GR, Chiche JD, Coopersmith CM, Hotchkiss RS, Levy MM, Marshall JC, Martin GS, Opal SM, Rubenfeld GD, van der Poll T, Vincent JL, Angus DC (2016). The Third International Consensus Definitions for Sepsis and Septic Shock (Sepsis-3). JAMA.

[13] Sessler CN, Gosnell MS, Grap MJ, Brophy GM, O’Neal PV, Keane KA, Tesoro EP, Elswick RK (2002). The Richmond Agitation-Sedation Scale: validity and reliability in adult intensive care unit patients. Am J Respir Crit Care Med.

[14] Oda S, Aibiki M, Ikeda T, Imaizumi H, Endo S, Ochiai R, Kotani J, Shime N, Nishida O, Noguchi T, Matsuda N, Hirasawa H (2014). The Japanese guidelines for the management of sepsis. J Intensive Care.

[15] Ritchie RF, Palomaki GE, Neveux LM, Navolotskaia O, Ledue TB, Craig WY (1999). Reference distributions for the negative acute-phase serum proteins, albumin, transferrin and transthyretin: a practical, simple and clinically relevant approach in a large cohort. J Clin Lab Anal.

[16] Kushimoto S, Gando S, Saitoh D, Ogura H, Mayumi T, Koseki K, Ikeda T, Ishikura H, Iba T, Ueyama M, Eguchi Y, Otomo Y, Okamoto K, Endo S, Shimazaki S (2008). Clinical course and outcome of disseminated intravascular coagulation diagnosed by Japanese Association for Acute Medicine criteria. Comparison between sepsis and trauma. Thromb Haemost.

[17] Szelenyi J, Kiss JP, Vizi ES (2000). Differential involvement of sympathetic nervous system and immune system in the modulation of TNF-α production by α_2_- and β-adrenoceptors in mice. J Neuroimmunol.

[18] Klimscha W, Tong C, Eisenach JC (1997). Intrathecal α_2_-adrenergic agonists stimulate acetylcholine and norepinephrine release from the spinal cord dorsal horn in sheep. An in vivo microdialysis study. Anesthesiology.

[19] Maes M, Lin A, Kenis G, Egyed B, Bosmans E (2000). The effects of noradrenaline and alpha-2 adrenoceptor agents on the production of monocytic products. Psychiatry Res.

[20] Lai YC, Tsai PS, Huang CJ (2009). Effects of dexmedetomidine on regulating endotoxin-induced up-regulation of inflammatory molecules in murine macrophages. J Surg Res.

[21] Zhang J, Wang Z, Wang Y, Zhou G, Li H (2015). The effect of dexmedetomidine on inflammatory response of septic rats. BMC Anesthesiol.

[22] Wu Y, Liu Y, Huang H, Zhu Y, Zhang Y, Lu F, Zhou C, Huang L, Li X, Zhou C (2013). Dexmedetomidine inhibits inflammatory reaction in lung tissues of septic rats by suppressing TLR4/NF-κB pathway. Mediat Inflamm.

[23] Qiao H, Sanders RD, Ma D, Wu X, Maze M (2009). Sedation improves early outcome in severely septic Sprague Dawley rats. Crit Care.

[24] Xiang H, Hu B, Li Z, Li J (2014). Dexmedetomidine controls systemic cytokine levels through the cholinergic anti-inflammatory pathway. Inflammation.

[25] Zamani MM, Keshavarz-Fathi M, Fakhri-Bafghi MS, Hirbod-Mobarakeh A, Rezaei N, Bahrami A, Nader ND (2016). Survival benefits of dexmedetomidine used for sedating septic patients in intensive care setting: a systematic review. J Crit Care.

[26] Gatta A, Verardo A, Bolognesi M (2012). Hypoalbuminemia. Intern Emerg Med.

[27] Chelazzi C, Villa G, Mancinelli P, De Gaudio AR Adembri C. (2015). Glycocalyx and sepsis-induced alterations in vascular permeability. Crit Care.

[28] Levi M, van der Poll T (2017). Coagulation and sepsis. Thromb Res.

[29] Patel P, Walborn A, Rondina M, Fareed J, Hoppensteadt D (2019). Markers of inflammation and infection in sepsis and disseminated intravascular coagulation. Clin Appl Thromb Hemost.

[30] Taniguchi T, Kurita A, Kobayashi K, Yamamoto K, Inaba H (2008). Dose- and time-related effects of dexmedetomidine on mortality and inflammatory responses to endotoxin-induced shock in rats. J Anesth.

[31] Otto GP, Sossdorf M, Claus RA, Rodel J, Menge K, Reinhart K, Bauer M, Riedemann NC (2011). The late phase of sepsis is characterized by an increased microbiological burden and death rate. Crit Care.

[32] Lobo SM, Lobo FR, Bota DP, Lopes-Ferreira F, Soliman HM, Melot C, Vincent JL (2003). C-reactive protein levels correlate with mortality and organ failure in critically ill patients. Chest.

[33] Ranzani OT, Prada LF, Zampieri FG, Battaini LC, Pinaffi JV, Setogute YC, Salluh JI, Povoa P, Forte DN, Azevedo LC, Park M (2012). Failure to reduce C-reactive protein levels more than 25% in the last 24 hours before intensive care unit discharge predicts higher in-hospital mortality: a cohort study. J Crit Care.

[34] Simon L, Gauvin F, Amre DK, Saint-Louis P, Lacroix J (2004). Serum procalcitonin and C-reactive protein levels as markers of bacterial infection: a systematic review and meta-analysis. Clin Infect Dis.

[35] Huang MY, Chen CY, Chien JH, Wu KH, Chang YJ, Wu KH, Wu HP. Serum procalcitonin and procalcitonin clearance as a prognostic biomarker in patients with severe sepsis and septic shock. Biomed Res Int. 2016. 10.1155/2016/1758501. 10.1155/2016/1758501 PMC4818793 27088084

[36] Oh TK, Song IA, Lee JH (2018). Clinical usefulness of C-reactive protein to albumin ratio in predicting 30-day mortality in critically ill patients: a retrospective analysis. Sci Rep.

