# Supplementary material for: Effect of dexmedetomidine on inflammation in patients with sepsis requiring mechanical ventilation: a sub-analysis of a multicenter randomized clinical trial
Source: Crit Care. 2020 Aug 10;24:493. doi: 10.1186/s13054-020-03207-8 (PMC7416813; doi:10.1186/s13054-020-03207-8)

Additional file 1

**Supplemental Methods**

**Study design**

To evaluate whether a sedation strategy with dexmedetomidine can improve clinical outcomes, we conducted the Dexmedetomidine for Sepsis in Intensive Care Unit (ICU) Randomized Evaluation (DESIRE) trial which was a multicenter, open-label randomized clinical trial in patients who had sepsis requiring mechanical ventilation [1]. The DESIRE trial was registered at Clinical Trials. gov (identifier: NCT 01760967). The trial was conducted in 8 ICUs in Japan. The first patient was enrolled in February 2013 and the trial was completed in January 2016. This study was approved by the institutional review boards of Wakayama Medical University and each participating institution. Written informed consent was obtained from the patient or patient’s family before randomization. Patients did not receive a stipend for participation in this trial. The co-primary outcomes of the trial were 28-day mortality and ventilator-free days over 28 days. Because duration of mechanical ventilation was highly influenced by mortality, we changed 28-days ventilator free days from ventilator days as primary endpoint. The 28-days ventilator free days was calculated by 28 days or living days minus day under mechanical ventilation after randomization. To estimate the sample size, we set 28-day survival would be 80% in DEX group and 60% in non-DEX group based on the results of MENDS randomized controlled trial, which showed that the 28-day survival was 84% among the group receiving dexmedetoidine and 59% among the control group [2]. 172 patients were needed to achieve 80% power with a 2-sided alpha level of 5%. A 15% dropout or withdrawal rate were estimated; and 200 patients were planned to enroll. Clinical outcomes were analyzed according to the intention-to-treat principle. All time-to-event data were censored at 28 days. Missing data were analyzed without imputation but there were no missing data of observations during the ICU stay.

We included the allocation variables of presence of emergency surgery, chronic obstructive pulmonary disease, and infection sites as covariates and study center were included as stratification variable to take into account for the differences between study centers.

All statistical analyses were conducted by independent stratification who was masked for allocation.

**Patients**

Patients who were 20 years old or older, had sepsis, and who ICU physician considered need mechanical ventilation for at least 24 hours were eligible for the study. Sepsis was defined as systemic inflammatory response syndrome due to infection [3]. Because fluid resuscitation and anti-sepsis treatments are necessary for acute pancreatitis (similar to an infection), cases of acute pancreatitis were included as an infection whereas cases of burns or heat stroke were not. Soft-tissue infection including necrotizing fasciitis and Fournier gangrene were considered to take unusually longer treatment for debridement in the ICU. Patients were excluded if they (1) had severe chronic liver disease (Child-Pugh B or C), (2) had acute myocardial infarction or severe heart failure (class 4 on the New York Heart Association functional classification system), (3) were drug dependence or alcoholism, or a psychological illness or severe cognitive dysfunction, (4) were pregnant or lactating or were allergic to dexmedetomidine.

**Randomization and Intervention**

Eligible patients were randomly assigned to receive either the sedation strategy with dexmedetomidine (DEX group) or the sedation strategy without dexmedetomidine (non-DEX group). Registration and data management were conducted using an electronic data capturing

System. Randomization was conducted by permutated block randomization stratified by study center, presence of emergency surgery, chronic obstructive pulmonary disease, and infection sites. The block size was four but not notified to physicians or investigators during the study.

Patients in DEX group received dexmedetomidine and analgesia constantly, and other sedatives were added as needed. Patients in non-DEX group received sedative drugs such as propofol, midazolam, and analgesia without dexmedetomidine. Controlled the sedation level according to the Richmond Agitation Sedation Scale (RASS) to achieve a score of 0 (calm) during the day and a score of -2 (lightly sedated) during the night in both groups [4]. Sedation was maintained throughout the duration of mechanical ventilation or as needed. The sedation protocols were also in compliance with the Clinical Practice Guidelines for Sustained Use of Sedatives and Analgesics in the Critically Ill Adult [5].

**Reference**

1. Kawazoe Y, Miyamoto K, Morimoto T, Yamamoto T, Fuke A, Hashimoto A, Koami H, Beppu S, Katayama Y, Itoh M, Ohta Y, Yamamura H (2017) Effect of Dexmedetomidine on Mortality and Ventilator-Free Days in Patients Requiring Mechanical Ventilation With Sepsis: A Randomized Clinical Trial. JAMA 317:1321-1328. https://doi.10.1001/jama.2017.2088
2. Pandharipande PP, Sanders RD, Girard TD, McGrane S, Thompson JL, Shintani AK, Herr DL, Maze M, Ely EW; MENDS investigators (2010) Effect of dexmedetomidine versus lorazepam on outcome in patients with sepsis: an a priori-designed analysis of the MENDS randomized controlled trial Crit Care 14:R38
3. Bone RC, Balk RA, Cerra FB, Dellinger RP, Fein AM, Knaus WA, Schein RM, Sibbald WJ (1992) Definitions for sepsis and organ failure and guidelines for the use of innovative therapies in sepsis. The ACCP/SCCM Consensus Conference Committee. American College of Chest Physicians/Society of Critical Care Medicine. Chest 101:1644-1655
4. Sessler CN, Gosnell MS, Grap MJ, Brophy GM, O’Neal PV, Keane KA, Tesoro EP, Elswick RK (2002) The Richmond Agitation-Sedation Scale: validity and reliability in adult intensive care unit patients. Am J Respir Crit Care Med 166:1338-1344
5. Barr J, Fraser GL, Puntillo K, Ely EW, Gélinas C, Dasta JF, Davidson JE, Devlin JW, Kress JP, Joffe AM, Coursin DB, Herr DL, Tung A, Robinson BR, Fontaine DK, Ramsay MA, Riker RR, Sessler CN, Pun B, Skrobik Y, Jaeschke R; American College of Critical Care Medicine (2013) Clinical practice guidelines for the management of pain, agitation, and delirium in adult patients in the intensive care unit. Crit Care Med 41:263-306

**Table S1: Scoring system for DIC**

|  | Score |
| --- | --- |
| Systemic inflammatory response syndrome criteria |  |
| ≥3 | 1 |
| 0-2 | 0 |
| Platelet count (10^9^/L) |  |
| <80 or 50% decrease within 24 hrs | 3 |
| ≥80 and <120 or >30% decrease within 24hrs | 1 |
| ≥120 | 0 |
| Prothrombin time (value of patient/ normal value) |  |
| ≥1.2 | 1 |
| <1.2 | 0 |
| Fibrin/fibrinogen degradation products (mg/L) |  |
| ≥25 | 3 |
| ≥10 and <25 | 1 |
| <10 | 0 |
| Diagnosis |  |
| Four points or more | DIC |

Abbreviations: DIC, disseminated intravascular coagulation

**Table S2: Changes in DIC and DIC-associated variables**

| Variable | DEX group | non-DEX group | P-value |
| --- | --- | --- | --- |
| PLT count ×10^9^, mean (SD) |  |  |  |
| Day 1 | 178 (114) | 163 (100) | 0.72 |
| Day 2 | 133 (92) | 127 (83) |  |
| Day 4 | 116 (94) | 104 (85) |  |
| Day 6 | 138 (99) | 128 (96) |  |
| Day 8 | 188 (125) | 178 (115) |  |
| Day 10 | 245 (159) | 222 (121) |  |
| Day 12 | 300 (196) | 253 (142) |  |
| Day 14 | 317 (193) | 299 (160) |  |
| PT-INR, mean (SD) |  |  |  |
| Day 1 | 1.29 (0.37) | 1.36 (0.50) | 0.03 |
| Day 2 | 1.38 (0.43) | 1.48 (0.95) |  |
| Day 4 | 1.18 (0.25) | 1.28 (0.55) |  |
| Day 6 | 1.16 (0.18) | 1.21 (0.44) |  |
| Day 8 | 1.18 (0.23) | 1.22 (0.31) |  |
| Day 10 | 1.17 (0.25) | 1.24 (0.40) |  |
| Day 12 | 1.14 (0.15) | 1.27 (0.41) |  |
| Day 14 | 1.13 (0.16) | 1.28 (0.54) |  |
| FDPs (mcg/mL), mean (SD) |  |  |  |
| Day 1 | 30.7 (43.6) | 51.7 (142.1) | 0.40 |
| Day 2 | 29.2 (33.0) | 42.5 (109.5) |  |
| Day 4 | 34.3 (48.5) | 29.8 (44.6) |  |
| Day 6 | 27.7 (29.0) | 35.7 (57.1) |  |
| Day 8 | 27.4 (26.5) | 30.8 (33.5) |  |
| Day 10 | 24.2 (23.5) | 24.2 (19.9) |  |
| Day 12 | 23.7 (26.0) | 20.3 (14.7) |  |
| Day 14 | 22.9 (24.7) | 19.1 (13.5) |  |
| ≥3 SIRS characteristics, n (%) |  |  |  |
| Day 1 | 71 (71) | 75 (74) | 0.15 |
| Day 2 | 52 (54) | 54 (56) |  |
| Day 4 | 26 (31) | 32 (37) |  |
| Day 6 | 16 (26) | 21 (31) |  |
| Day 8 | 11 (20) | 18 (29) |  |
| Day 10 | 9 (18) | 16 (32) |  |
| Day 12 | 7 (19) | 11 (26) |  |
| Day 14 | 4 (12) | 7 (19) |  |
| DIC, n (%) |  |  |  |
| Day 1 | 40 (40) | 42 (42) | 0.49 |
| Day 2 | 50 (52) | 57 (59) |  |
| Day 4 | 50 (59) | 48 (56) |  |
| Day 6 | 28 (46) | 34 (50) |  |
| Day 8 | 20 (38) | 29 (48) |  |
| Day 10 | 18 (37) | 18 (37) |  |
| Day 12 | 11 (31) | 12 (30) |  |
| Day 14 | 7 (21) | 6 (17) |  |

Abbreviations: DEX, dexmedetomidine DIC, disseminated intravascular coagulation; SD, standard deviation; PLT, platelet; PT-INR, prothrombin time-international ratio; FDPs, fibrin/fibrinogen degradation products; SIRS, systemic inflammatory response syndrome

**Figure S1. Flow of Participants in the DESIRE trial**


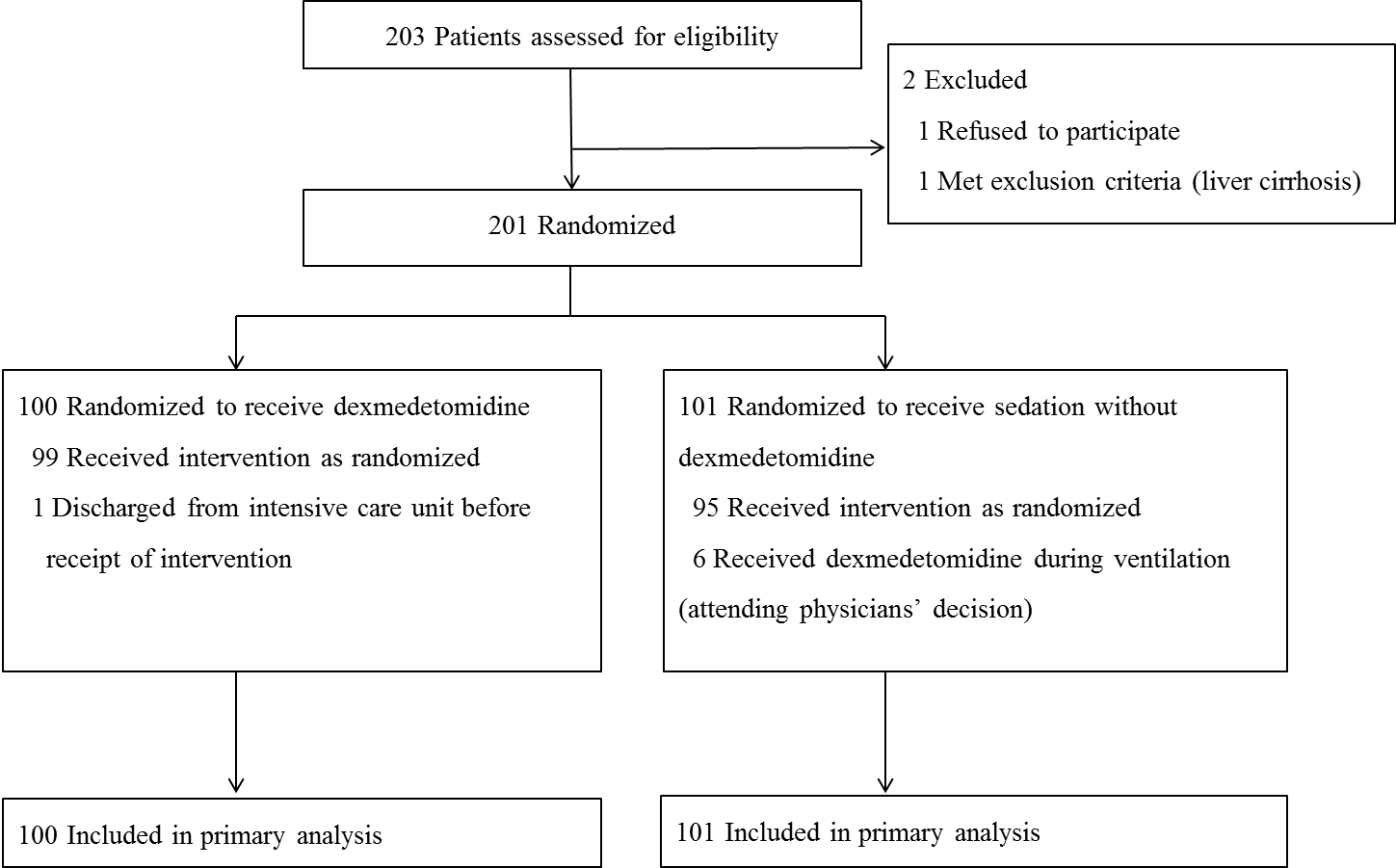

Supplement: Supplementary file 1 — Additional file 1: Supplemental Methods. Table S1. Scoring system for DIC. Table S2. Changes in DIC and DIC-associated variables. Figure S1. Flow of Participants in the DESIRE trial. [file 13054_2020_3207_MOESM1_ESM.docx]
